# Glomerular Damage in Trichloroethylene-Sensitized Mice: Targeting Cathepsin L-Induced Hyperactive mTOR Signaling

**DOI:** 10.3389/fphar.2021.639878

**Published:** 2021-07-29

**Authors:** Feng Wang, Yuying Dai, Meng Huang, Chenchen Zhang, Liping Huang, Hui Wang, Liangping Ye, Qifeng Wu, Xuejun Zhang, Qixing Zhu

**Affiliations:** ^1^ Department of Dermatology, The Second Hospital of Anhui Medical University, Hefei, China; ^2^ Key Laboratory of Dermatology, Ministry of Education, The First Affiliated Hospital of Anhui Medical University, Hefei, China; ^3^ Department of Occupational Health and Environmental Health, School of Public Health, Anhui Medical University, Hefei, China; ^4^ Department of Dermatology, The First Affiliated Hospital of Anhui Medical University, Hefei, China; ^5^ Poison Control Center, Guangdong Province Hospital for Occupational Disease Prevention and Treatment, Guangzhou, China

**Keywords:** trichloroethylene, podocyte, apoptosis, mTOR, cathepsin L

## Abstract

Trichloroethylene (TCE) is a serious health hazard for workers with daily exposure, causing occupational medicamentosa-like dermatitis due to TCE (OMDT) and glomerular damage. Recent studies suggest that mTORC1 signaling is activated in various glomerular disorders; however, the role of mTORC1 signaling in TCE-induced glomerular damage remains to be explored. In the present study, 6 OMDT patients were enrolled and a TCE-sensitized mouse model was established to investigate molecular mechanisms underlying the glomerular damage associated with OMDT. Glomerular damage was assessed by levels of urine nephrin, H&E staining, and renal function test. Ultrastructural change of podocyte was investigated by transmission electron microscopy. The podocyte-related molecules including nephrin, α-actinin-4, and integrin β1 were visualized by immunofluorescence. The activation of mTORC1 signaling was confirmed by Western blot. Glomerular apoptosis was examined by the TUNEL test and Western blotting. Expression and location of cathepsin L (CTSL) were assessed by RT-PCR and immunofluorescence. Our results showed that TCE sensitization caused damage to glomerular structural integrity and also increased the activation of mTORC1 signaling, which was accompanied by podocyte loss, hypertrophy, and glomerular apoptosis. Importantly, we also found that over-expressed CTSL was mainly located in podocyte and CTSL inhibition could partially block the activation of mTORC1 signaling. Thus, our findings suggested a novel mechanism whereby hyperactive mTOR signaling contributes to TCE sensitization–induced and immune-mediated glomerular damage via CTSL activation.

## Introduction

Trichloroethylene (TCE) is a volatile and chlorinated organic solvent widely used in industrial settings such as metal degreasing, parts cleaning, and refrigerants manufacturing (Byrum et al., 2019; Pan et al., 2019). Over the past decades, the extensive use of TCE has caused more than 50 million pounds annually released into the environment in the United States and even heavier in China (Huang et al., 2014). There are more than 20,000 workers that are exposed to TCE every year and TCE has become an intractable environment concern, and a serious health hazard to the public in China (Huang et al., 2014). The International Agency for Research on Cancer (IARC) has determined TCE as Group 1 carcinogen to humans (IARC, 2014). Recent epidemiological studies show that TCE exposure was closely linked to cancers such as renal cancer and non-Hodgkin lymphoma (Guha et al., 2012; Lee et al., 2019), and trichloroethylene hypersensitivity syndrome (THS) (Li et al., 2019).

THS, also called occupational medicamentosa-like dermatitis due to trichloroethylene (OMDT) in China, is a severe hypersensitivity reaction, after TCE skin contact or inhalation in occupational environment (Ministry of Health, 2006). OMDT is a T-cell–mediated type IV hypersensitivity reaction, presented with erythema, rash, blisters, and systems damage (Lin et al., 2019). However, the allergy theory alone could not fully explain the complicated pathogenesis of OMDT, including the severe hepatitis and renal complications. Studies in humans and experimental animals confirmed that a mixed-type allergic response involving cellular and humoral immunity (Huang et al., 2015) contributes to the multiple organ injuries in OMDT. Glomerulus is responsible for filtering blood under physiological conditions and is a primary target of various physical and chemical factors in kidney disorders. Our previous studies reported pathological changes of glomerulus with impaired filtration function in TCE-induced immune kidney disorder with unknown mechanisms (Wang et al., 2019b).

Podocytes are terminally differentiated epithelial cells, covering the surface of glomerular basement membrane (GBM) via foot process extensions (Sakhi et al., 2019). The special foot processes form the slit diaphragm (SD), which is the ultimate filtration barrier of glomerulus (Maeda et al., 2018). In addition, to accomplish the normal filtering task, various molecules such as nephrin and podocin, integrin β1, and α-actinin-4 work together as an interacting network and ensure the normal podocyte’s cytoskeleton and glomerular integrity (Sawada et al., 2016; Rinschen et al., 2017). However, this dynamic balance of the molecular network is easily broken and the imbalance leads to renal dysfunction and even proteinuria (Kriz and Lemley, 2015).

In general, mammalian target of rapamycin (mTOR) is a highly conserved serine/threonine kinase that plays an important role for cell proliferation, autophagy, and apoptosis (Fantus et al., 2016; Cui et al., 2020). mTOR contains two catalytic subunits such as mTOR complex 1 (mTORC1) and mTOR complex 2, and mTORC1 signaling is capable to regulate the podocyte size, implicated in a variety of kidney disorders (Zschiedrich et al., 2017; Puelles et al., 2019). However, whether the activation of mTORC1 signaling is involved in the podocyte damage in TCE sensitization or not is still unknown. Here, we employed a mouse model of TCE skin sensitization to uncover the detailed role of mTORC1 signaling in TCE-induced glomerular damage.

## Materials and Methods

### Reagents

TCE (99.9% purity), Freund's complete adjuvant (FCA, composition: 85% Drakeol 6 VR (mineral oil); 15% mannide monooleate (Arlacel A); 20 mg *Mycobacterium*), and *In Situ* Cell Death Detection Kit were purchased from Sigma-Aldrich (St. Louis, Missouri, United States). Acetone and olive oil were purchased from Shanghai Chemical Reagent Company (Shanghai, China). The human nephrin DuoSet ELISA kit was obtained from R&D System (Minneapolis, United States). TRIzol and RevertAid First Strand cDNA Synthesis Kit were obtained from ThermoFisher Scientific (MA, United States). The antibodies against nephrin, α-actinin-4, cathepsin L, IgG H&L AlexaFluor^®^ 488, and AlexaFluor^®^ 594 were purchased from Abcam (Cambridge, United Kingdom). The antibody against podocin was purchased from Santa Cruz Biotechnology (Dallas, TX, United States). The antibody against integrin β1 was purchased from Affinity Biosciences (OH, United States). Antibodies against mTOR, p-mTOR, 4EBP1, p-4EBP1, p70S6K, p-p70S6K, Bax, Bcl-2, caspase-3, and GAPDH were purchased from Cell Signaling Technology (Beverly, MA, United States). 4′, 6-diamidino-phenylindole dihydrochloride (DAPI) was purchased from Solarbio Life Sciences (Beijing, China). Rapamycin and Z-Phe-Tyr-CHO were supplied by Selleck and Santa Cruz Biotechnology, respectively.

### Ethics

The experimental design and all protocols were approved by the Biomedical Ethics Committee of Anhui Medical University (No. 20160216) and Experimental Animal Ethics Committee of Anhui Medical University (No. LLSC20160312) and followed the Declaration of Helsinki principles. All participants were informed about the objective and methods of this study before signing the informed consent. The mouse experiments were performed in accordance with NIH guidelines for care and use of laboratory animals.

### Study Participants

Newly diagnosed OMDT patients and healthy controls were recruited from January 2017 to December 2019 at Poison Control Center of Guangdong Province Hospital for Occupational Disease Prevention and Treatment. The OMDT was diagnosed based on the history of TCE exposure, fever, skin lesions, and multisystem damage according to the Chinese National Diagnostic Criteria (GBZ 185-2006). Urine was collected before and after clinical treatment for nephrin examination. The baseline data of OMDT patients in the present study is summarized in Table 2.

### Mice and TCE Exposure

**FIGURE 1:**
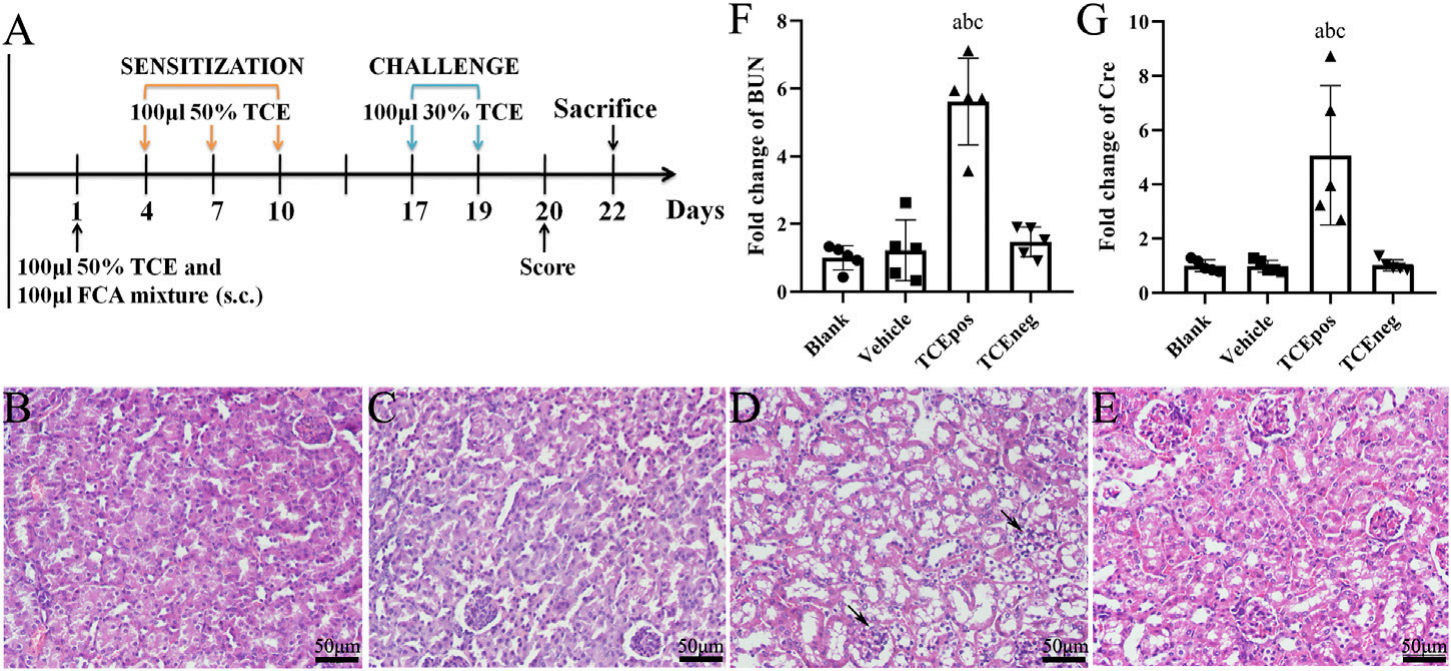
TCE sensitization caused the glomerular damage with filtering dysfunction. **(A)** Flow diagram of BALB/c mouse model of TCE skin sensitization. **(B-E)** Representative pictures of H&E staining (*n* = 5 per group in blank control group **(B)**, vehicle control group **(C)**, TCEpos group **(D)**, and TCEneg group **(E)**. Black arrows show glomerular cell edema (magnification, × 400). **(F, G)** Relative expression of BUN and Cre in TCE-treated mice (*n* = 5). Data are presented as mean ± SD and determined by one-way ANOVA. ^a^ *P* < 0.05, vs. blank control group; ^b^ *P*<0.05, vs. vehicle control group; ^c^ *P*<0.05, vs. TCEneg group. Scale bars: 50 µm.

A total of 140 female BALB/c mice (6–8 weeks-old and 18–20 g of weight) were obtained from the Experimental Animal Center of Anhui Medical University. Mice were housed in specific pathogen free environment with food and water *ad libitum*. The feeding conditions were manually set as follows: 22.5 ± 0.5°C temperature, 50 ± 5% humidity, and 12 h light/dark cycle. Mice were acclimated for 7 days before experimental initiation. The mouse model of TCE skin sensitization was established according to previous studies (Wang et al., 2015; Wang et al., 2019a; Wang et al., 2019b). Briefly, sensitization phase and challenge phase were conducted sequentially after the initial administration of 100 μL 50% TCE mixture and equal FCA on the 1st day. The sensitization phase was carried out by spread of 100 μL 50% TCE mixture (TCE: olive oil: acetone = 5: 2: 3, v/v/v) on the back skin every 3 days for three times until the 10th day. Then, the challenge phase was performed 2 times by topical application of 100 μL 30% TCE mixture (TCE: olive oil: acetone = 3: 2: 5, v/v/v) on the same area on the 17th day and 19th day (Figure 1A). Mice with physiological saline or vehicle (the same proportions of olive oil and acetone without TCE) treatment were considered as controls. Additionally, to explore the mTOR signaling, rapamycin and Z-Phe-Tyr-CHO were applied by intraperitoneal injection at a dose of 4 mg/kg and 10 mg/kg, respectively. The details of the group design are listed in Table 1.

**TABLE 1:** Sensitization rate and kidney impairment incidence in mice.

Groups	Mice (n)	Score				Rate (%)	
		0	1	2	3	Sensitization	Kidney impairment
Blank control	10	0	0	0	0	0.0	0
Vehicle control	10	0	0	0	0	0.0	0
TCE treatment	40	25	9	4	2	37.5 (15/40)	35.0 (14/40)
TCEpos	15		9	4	2		
TCEneg	25	25					
RAPA + TCE treatment	40	26	11	3	0	35.0 (14/40)	15.0 (6/40)
RAPA + TCEpos	14		11	3	0		
RAPA + TCEneg	26	26					
CTSLinh + TCE treatment	40	27	9	4	0	32.5 (13/40)	12.5 (5/40)
CTSLinh + TCEpos	13		9	4	0		
CTSLinh + TCEneg	27	27					

CTSL, cathepsin L; inh, inhibitor; TCE, trichloroethylene; pos, positive; neg, negative; RAPA, rapamycin.

Twenty four hours after the last challenge, the cutaneous allergic reactions were scored by the severity of erythema and edema: 0, absolutely normal; 1, scattered or mild erythema; 2, moderate and diffuse erythema; 3, intensive erythema and swelling. Score ≥ 1 is identified as positive sensitization and score = 0 means negative sensitization. Mice were euthanized by CO_2_ and sacrificed, 72 h after the last challenge. Blood was taken from ophthalmic venous plexus and centrifuged to separate serum from blood cells at 4°C, 1000 × *g* for 30 min. The collected kidneys were embedded in paraffin for histological examination, or embedded in the optimal freezing medium (OCT) for immunofluorescence and glomeruli were isolated from fresh kidney tissues.

### ELISA

Levels of nephrin in the urine of OMDT patients were analyzed by ELISA following the manufacturer’s instructions. In brief, the plate was coated with the capture antibody and was blocked by reagent diluent. The urine samples and prepared standards were added into well plates for two hours incubation. Then, diluted detection antibody, streptavidin-HRP working dilution, substrate solution, and stop solution were added to each well sequentially. Finally, the optical density was determined by a microplate reader (Bio-Tek, μQuant) and the concentration of urine nephrin was calculated by the standard curve.

### Histological Examination

Fresh kidneys were removed and fixed with 10% formaldehyde for about 48 h. Then, the kidneys were embedded in paraffin and sectioned at 5 μm thickness. After standard dewaxing and hydration, sections were stained by hematoxylin/eosin staining (H&E staining) to analyze the morphological changes in glomerulus.

### Transmission Electron Microscope Detection

To assess the ultrastructural changes of podocyte, the fresh kidney cortex was cut into 1 mm^3^ and fixed in 2.5% glutaraldehyde for 6–12 h. After postfixation in 2% osmic acid solution for 2 h, the samples were dehydrated by graded ethanol (50% ethanol, 70% ethanol, and 90% ethanol) and propylene oxide, and embedded in Epon 812. Then, the samples were sectioned at 60 nm thickness and scanned in a transmission electron microscope (JEM-1230, Japan).

### Renal Function Assessment

Blood urea nitrogen (BUN) and serum creatinine (Cre) are classical and conventional indicators to reflect the renal function in clinical practice. In this study, BUN and Cre were measured by commercial assay kits (Nanjing Jiancheng Bioengineering Institute, China). The optical density of BUN and Cre was recorded at 640 and 546 nm respectively in a microplate reader (Bio-Tek, μQuant). Then, the levels of BUN and Cre were calculated according to the standard curve handled simultaneously with the samples.

### TUNEL Assays

TUNEL assays were performed by *In Situ* Cell Death Detection Kit, POD (Roche). Briefly, the sections were covered with proteinase K working fluid for 30 min after routine dewaxing and hydration. Then, the prepared TUNEL reaction mixture was added to the sections in a dark humid chamber for 60 min. After PBS rinse for 3 times, a drop of PBS was added onto the sections to count the apoptotic cells under fluorescence microscope. Next, 50 μL converter-POD was covered on the sections in a dark humid chamber at 37°C for 30 min. DAB working fluid was used to stain the apoptotic cells counterstained with hematoxylin. The counting and analysis were performed under an optical microscope and the representative fields were collected simultaneously.

### Immunohistochemistry and Immunofluorescence Examination

Fresh kidney tissues were embedded in OCT compound. The tissues were cut into of 5 μm thickness frozen sections in a Leica CM1850 cryostat (Wetzlar, Germany) and the sections were fixed with precooled acetone for 5 min. Next, 0.3% Triton X-100 was covered on the sections for 30 min, followed by blocking with goat serum working fluid for 2 h at room temperature. The primary antibodies including anti-CTSL, anti-podocin (Diluted at 1:1000), anti-nephrin, anti-integrin β1 (Diluted at 1:200), and anti–α-actinin-4 were then incubated with the sections overnight at 4°C. Then, the sections were taken out and rewarmed to 37°C for 30 min, before linking to fluorescein labeled secondary antibodies: goat anti-rabbit IgG H&L (Diluted at 1:200) or goat anti-mouse IgG H&L (Diluted at 1:400). Two hours later, the sections were washed with PBS and the nuclei were counterstained with DAPI for 15 min. The localization and expression of marker proteins were observed under an inverted fluorescence microscope (Olympus, IX73, Japan) with appropriate excitation and emission filters.

### Glomeruli Isolation

After skin sterilization via 75% alcohol and ligation of superior mesenteric artery, thoracic aorta, the distal abdominal aorta, and distal inferior vena cava were dissected. A capillary was inserted into the middle of the abdominal aorta and precooled sterile PBS was pumped into kidney. After the residual blood in kidney was removed, 4 × 10^7^ magnetic beads in 20 ml normal saline were slowly pumped into kidney via the inserted capillary. Kidney was removed and cut into 1 mm^3^ small pieces for digestion using 1 mg/ml collagenase A. Then, the fluid was filtrated by a 100-μm cell strainer for 2 times. The collected suspension was centrifuged at 200 × *g* for 5 min and the sedimentation was resuspended with PBS. Finally, glomeruli were collected by a magnetic particle concentrator to extract total proteins.

### RT-PCR

The total RNA was extracted from glomerulus by TRIzol and cDNA was synthesized by RevertAid First Strand cDNA Synthesis Kit. The process of PCR was accomplished with SYBR Green I Master under LightCycler 480 system. The primer sequences of *cathepsin L* and *GAPDH* mRNA used here are listed as follows: *cathepsin L*, forward, 5’-CCC TAT GAA GCG AAG GAC GG-3’, reverse, 5’-CTG GAG AGA CGG ATG GCT TG-3’; *GAPDH*, forward, 5’-CCC TTA AGA GGG ATG CTG CC-3’, reverse, 5’-TAC GGC CAA ATC CGT TCA CA-3’.

### Western Blot Analysis

Total proteins were extracted from glomerulus by lysis buffer and the protein concentration was detected by using the BCA assay. The final concentration of protein samples was diluted to 10 mg/ml mixed with loading buffer. A total of 10 μL protein sample was added to the prepared gels and separated by SDS-PAGE. The proteins were then transferred to a PVDF membrane (0.45 μm, Millipore, United States), followed by blocking in 5% skim milk powder for 2 h at room temperature. The membranes were incubated at 4°C overnight with the primary antibodies against mTOR, phospho-mTOR, Akt, phospho-Akt, Bax, Bcl-2, caspase-3, β-actin, and GAPDH (diluted at 1:1000). Next, the membrane was washed by PBST and incubated with goat anti-rabbit IgG antibody (Diluted at 1:100000) or goat anti-mouse IgG antibody (Diluted at 1:10000) for 2 h at room temperature. Proteins were assessed by WesternBright ECL HRP substrate kit (Advansta, 171005-80, United States) in a chemiluminescence system (CLiNX ChemiScope 6000 Touch, Shanghai, China).

### Statistical Analysis

Statistical analysis was performed using GraphPad Prism software (version 6, San Diego, CA). Data were presented as mean ± SD. The paired *t*-test was carried out to compare the difference of nephrin in urine before and after clinical treatment. The χ^2^ test was used to compare the difference of mice sensitization rate and the incidence of kidney impairment. One-way ANOVA followed by Tukey’s or Bonferroni’s test was applied to compare statistical differences between multiple groups. *P*-value < 0.05 was considered statistically significant and *P*-value < 0.01 was also displayed in the results.

## Results

### Sensitization Rate and Occurrence of Kidney Impairment in Mice

As shown in Table 1, a total of 42 mice with mild or severe skin erythema and/or edema displayed a positive sensitization and the overall sensitization rate (excluding blank control and vehicle control group) was 35.0%. No visible skin lesions were found in mice from blank control group and vehicle control group. According to the cutaneous reaction and pharmacologic pretreatment, mice were grouped as follows: blank control group (*n* = 10), vehicle control group (*n* = 10), TCE sensitization positive subgroup (TCEpos, *n* = 15) and TCE sensitization negative subgroup (TCEneg, *n* = 25), TCE sensitization positive subgroup with rapamycin (mTOR inhibitor) pretreatment (RAPA + TCEpos, *n* = 14), TCE sensitization negative subgroup with rapamycin pretreatment (RAPA + TCEneg, *n* = 26), TCE sensitization positive subgroup with Z-Phe-Tyr-CHO (cathepsin L inhibitor) pretreatment (CTSLinh + TCEpos, *n* = 13), and TCE sensitization negative subgroup with Z-Phe-Tyr-CHO pretreatment (CTSLinh + TCEneg, *n* = 27).

H&E staining showed that 14 sensitization positive mice with renal structural changes were found in TCE treatment group whereas the number were dropped to 6 and 5 in RAPA + TCE treatment group and CTSLinh + TCE treatment group, respectively. The incidence of kidney damage in TCE treatment group was significantly higher than RAPA + TCE treatment group (15.0%) and CTSLinh + TCE treatment group (12.5%). These results showed that inhibition of mTOR and CTSL reduced TCE-induced sensitization, and suggest that mTOR and CTSL are involved in TCE-induced skin sensitization.

### TCE Sensitization Caused the Glomerular Damage With Filtration Dysfunction

OMDT is a rare but severe disorder with a prevalence of less than 1% among TCE-exposed workers (Lin et al., 2019). Six OMDT patients, including 2 females and 4 males, and 10 health controls were recruited in our study. Increased levels of serum BUN, urine albumin, and urine erythrocyte were found in several participants. The baseline data and kidney damage–related index was listed in Table 2.

**TABLE 2:** The baseline data of OMLDT patients in the present study.

	Gender	Ethnicities	Age	BMI	Pre-existing renal conditions	Comorbidities	Latent period (days)	Renal dysfunction
Case 1	Female	Han	29	17.9	No	No	38	NO
Case 2	Male	Han	18	22.3	No	No	32	NO
Case 3	Male	Han	21	24.4	No	No	37	NO
Case 4	Female	Han	19	24.5	No	No	30	YES (BUN 10.91)
Case 5	Male	Han	22	28.4	No	No	18	YES (BUN 9.7)
Case 6	Male	Han	18	17.1	No	No	27	YES (PRO +, BLD +)

BLD, urinary blood; BMI, body mass index; BUN, blood urea nitrogen (mmol/L); PRO, urinary protein.

In mice, H&E staining showed glomerular cell edema with or without inflammatory cell infiltration in TCEpos group. In contrast, no detectable structural changes were found in blank control group, vehicle control group, and TCEneg group (Figures 1B–E). Compared to vehicle control group, the levels of both BUN and Cre were increased in TCEpos group (*p* < 0.05), whereas no significant differences were found among blank control group, vehicle control group, and TCEneg group (*p* > 0.05) (Figures 1F,G). These data demonstrated TCE sensitization-specific glomerular structural and functional damage in both human and mouse model.

### Podocyte Morphological Changes Involved in TCE-Induced Glomerular Damage

**FIGURE 2:**
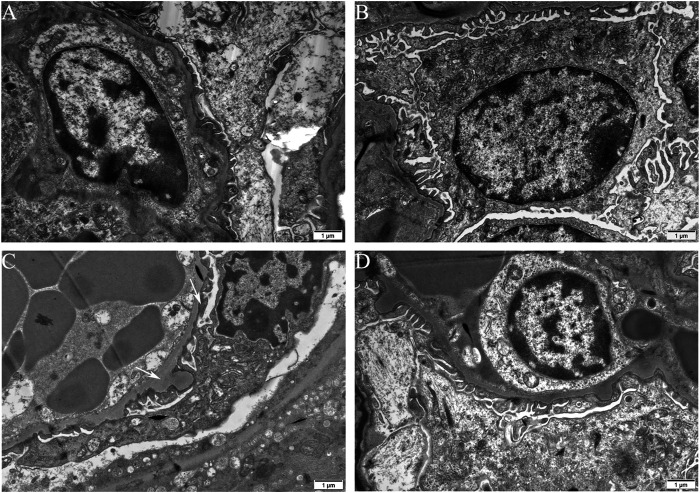
Podocyte morphological changes involved in TCE-induced glomerular damage. Representative observations in transmission electron microscopy (*n* = 5 per group, magnification, × 15000) of mice glomerulus from blank control group **(A)**, vehicle control group **(B)**, TCEpos group **(C)**, TCEneg group **(D)**. White arrows show podocyte hypertrophy with thickened glomerular basement membrane, fusion of foot processes in TCEpos group. Scale bars: 1 µm.

To estimate the ultrastructural changes in podocytes, transmission electron microscopy for mice was performed. The observations showed a well-distributed glomerular basement membrane and an orderly arranged foot process in blank control group, vehicle control group, and TCEneg group. However, podocyte hypertrophy with thickened glomerular basement membrane and fusion of foot processes were found in TCEpos group (Figure 2). In summary, these findings suggest that podocyte damage occurred in TCE-induced glomerular disorders.

### Loss of Podocytes Occurred in OMDT Patients and TCE-Sensitized Mice

**FIGURE 3:**
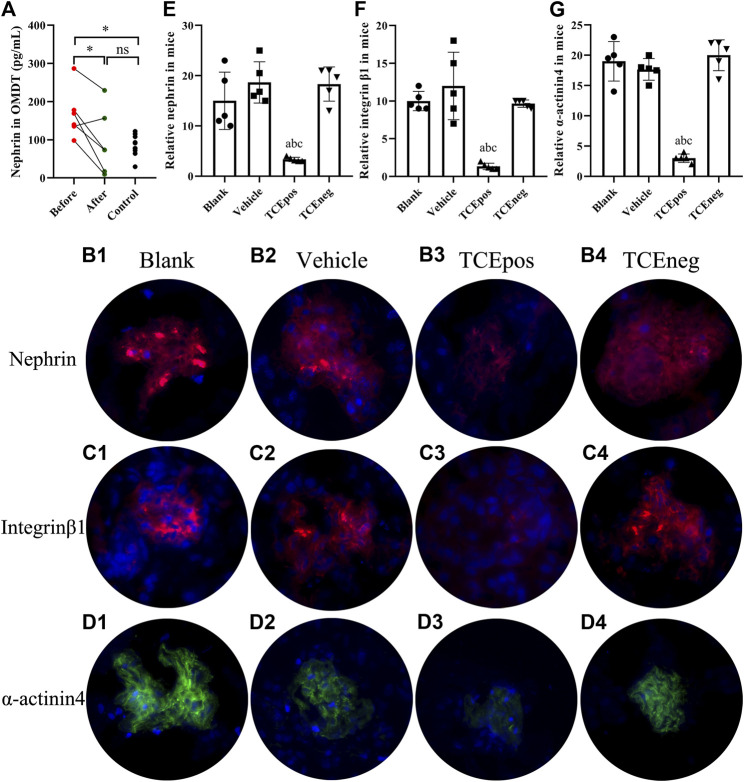
Loss of podocytes occurred in OMDT patients and TCE-sensitized mice. **(A)** Expression of urine nephrin before and after clinical treatment in OMDT patients (*n* = 6). **(B–D)** Representative results of immunofluorescence test in mice (*n* = 5 per group). **(B1–B4)** Expression of nephrin in blank control group **(B1)**, vehicle control group **(B2)**, TCEpos group **(B3)**, and TCEneg group **(B4)**. **(C1–C4)** Expression of integrin β1 in blank control group **(C1)**, vehicle control group **(C2)**, TCEpos group **(C3)**, and TCEneg group **(C4)**. **(D1–D4)** Expression of α-actinin-4 in blank control group **(D1)**, vehicle control group **(D2)**, TCEpos group **(D3)**, and TCEneg group **(D4)**. **(E-G)** The fluorescence intensity of nephrin, integrin β1, and α-actinin-4 calculated in Image J software. Data are presented as mean ± SD and determined by paired *t*-test or one-way ANOVA, *p < 0.05, ^ns^ *P*>0.05; ^a^ *P* < 0.05, *vs.* blank control group; ^b^ *P*<0.05, *vs.* vehicle control group; ^c^ *P*<0.05, *vs.* TCEneg group.

To assess the loss of podocyte in OMDT, nephrin, a specific marker of podocyte, was determined in urine before and after clinical treatment. The levels of nephrin in urine were significantly decreased after clinical treatment. Compared to the healthy control, the level of nephrin in OMDT patients before clinical treatment was increased and back to normal range after clinical treatment (*p* < 0.05, Figure 3A). In mice, podocytes were also lost in TCEpos group, presented as decreased levels of nephrin (Figures 3B,E). These data suggest that loss of podocytes contributes to the glomerular structure damage in OMDT.

Of importance, integrin β1 and α-actinin-4 are the key constitutive proteins of podocyte. Integrin β1 is a molecule for adhesion of podocytes to glomerular basement membrane and α-actinin-4 is crucial to sustain the normal cytoskeleton of podocyte. Therefore, we also measured the levels of integrin β1 and α-actinin-4 in mice glomerulus by immunofluorescence test with recorded fluorescence intensity in ImageJ software. Our results revealed that the expression of both α-actinin-4 and integrin β1 was decreased in glomerulus from TCEpos group than vehicle control group (*p* < 0.05). No statistical differences were found among blank control group, vehicle control group, and TCEneg group (*p* > 0.05) (Figures 3C,D,F,G).

### Glomerular Apoptosis Participated in TCE-Induced Kidney Disorder

**FIGURE 4:**
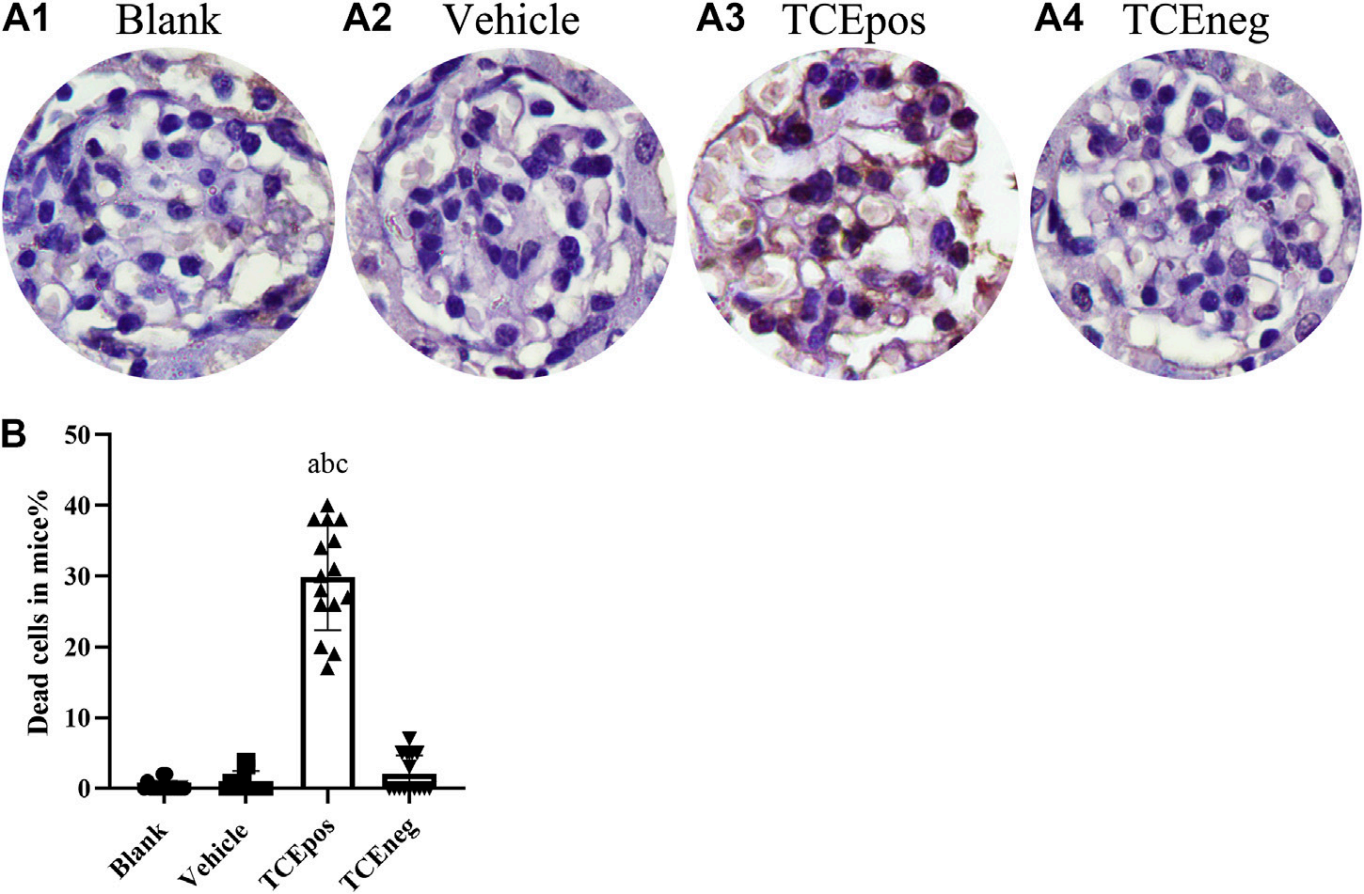
An increase proportion of dead cells in TCE-induced kidney glomerular damage **(A)** Representative photographs of TUNEL staining in blank control group **(A1)**, vehicle control group **(A2)**, TCEpos group **(A3)**, and TCEneg **(A4)**. **(B)** The proportion of TUNEL staining positive cells in TCE-treated mice. Note: the brown of nuclei indicates the positive staining. Data are presented as mean ± SD and determined by one-way ANOVA, ^a^ *P* < 0.05, vs. blank control group; ^b^ *P*<0.05, vs. vehicle control group; ^c^ *P*<0.05, vs. TCEneg group.

Considering that cell apoptosis was one of the common reasons of podocyte hypertrophy and glomerular integrity damage (Priante et al., 2019), we assessed the glomerular apoptosis by TUNEL staining. The proportion of glomerular dead cells in TCEpos group was significantly elevated compared with TCEneg group, vehicle control group, and blank control group (both *p* < 0.05) (Figure 4), suggesting increased cell death via apoptosis by TCE sensitization.

**FIGURE 5:**
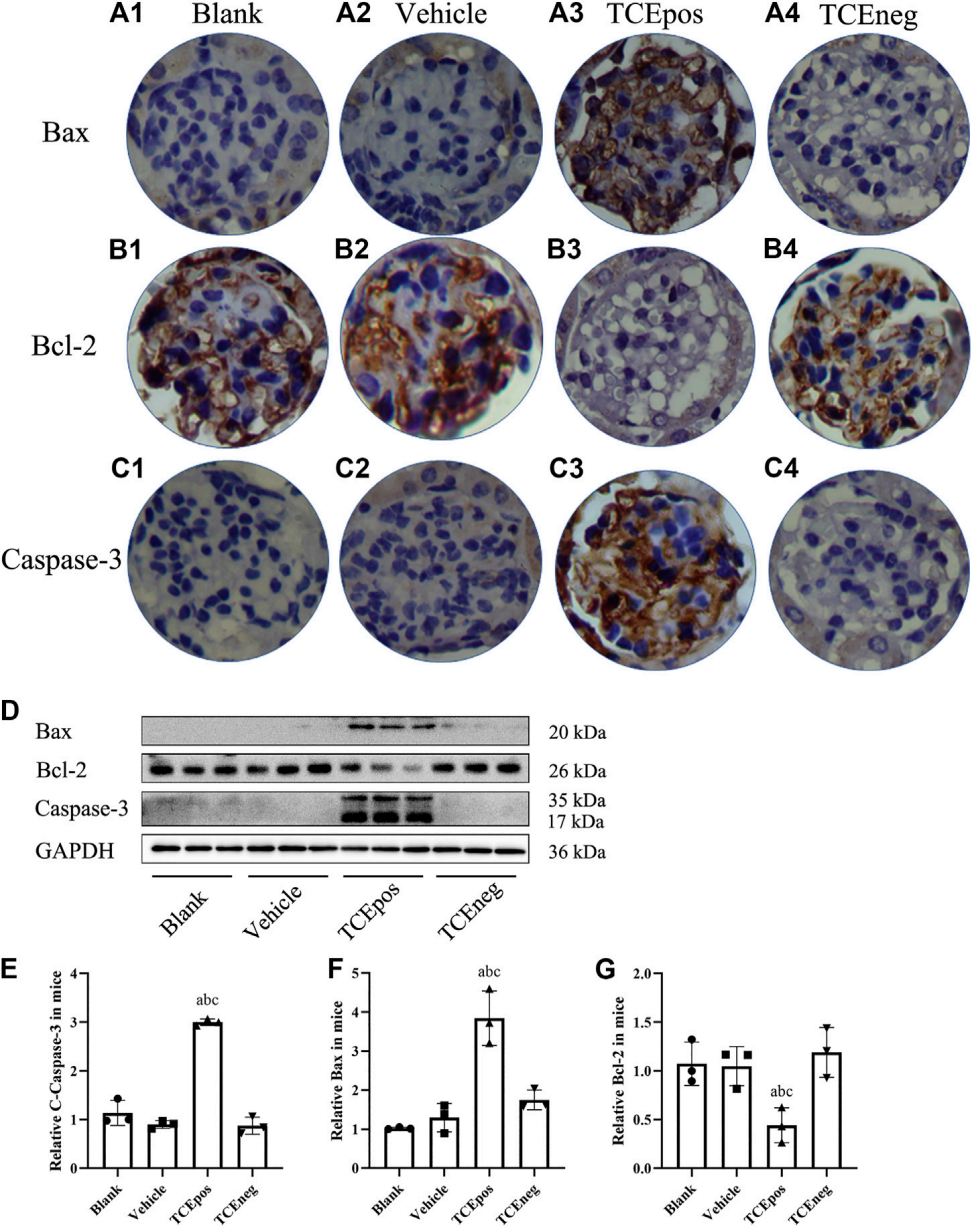
Activation of Bax/Bcl-2/caspase-3 pathway in TCE-induced glomerular damage. **(A1–A4)** Expression of proapoptotic molecule Bax; **(B1–B4)** expression of anti-apoptotic molecule Bcl-2; **(C1–C4)** expression of proapoptotic molecule caspase-3 in blank control group, vehicle control group, TCEpos group, and TCEneg group (*n* = 5 per group). **(D)** Western blot bands of Bax, Bcl-2, and caspase-3 in blank control group, vehicle control group, TCEpos group, and TCEneg group (*n* = 3 per group). **(E–G)** Relative expression of Bax, Bcl-2, and caspase-3 calculated by gray values in image J software in above groups. Data are presented as mean ± SD and determined by one-way ANOVA, ^a^ *P* < 0.05, vs. blank control group; ^b^ *P*<0.05, vs. vehicle control group; ^c^ *P*<0.05, vs. TCEneg group.

To further evaluate the participation of glomerular apoptosis, we tested the expression of Bax/Bcl-2/caspase-3, which is widely recognized as an apoptosis pathway (Ma et al., 2020). We showed that the pro-apoptotic molecules Bax and caspase-3 were elevated whereas the anti-apoptotic molecule Bcl-2 was decreased in TCEpos group compared with TCEneg group (*p* < 0.05). No significant differences were found among blank control, vehicle group, and TCEneg group (*p* > 0.05) (Figure 5).

### Hyperactive mTORC1 Pathway Was Involved in TCE-Induced Glomerular Damage

**FIGURE 6:**
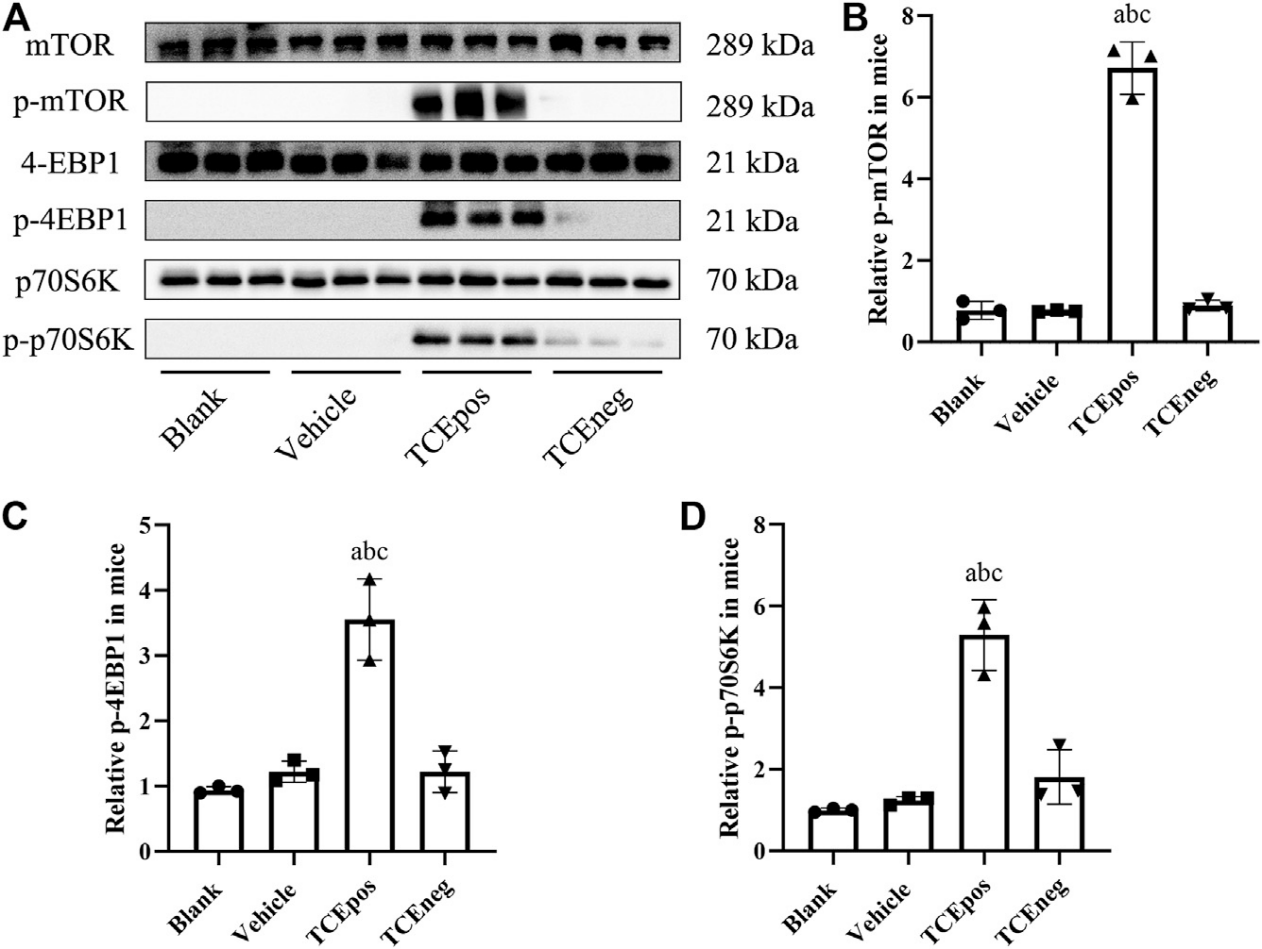
Hyperactive mTORC1 pathway was involved in TCE-induced glomerular damage. **(A)** Western blot bands of mTORC1 signaling molecules, mTORC1, p-mTORC1, 4EBP1, p-4EBP1, p70S6K, and p-p70S6K, in blank control group, vehicle control group, TCEpos group, and TCEneg group (*n* = 3 per group) **(B–D)** The relative expression of p-mTOR **(B)**, p-4EBP1 **(C)**, and p-p70S6K **(D)** calculated by gray values in ImageJ software in above groups. Data are presented as mean ± SD and determined by one-way ANOVA, ^a^ *P* < 0.05, vs. blank control group; ^b^ *P*<0.05, vs. vehicle control group; ^c^ *P*<0.05, vs. TCEneg group.

The mouse glomeruli were isolated to evaluate the levels of key phosphorylated (p-) proteins involved in the mTORC1 signaling activation. Compared to TCEneg group, the relative expression of mTORC1 signaling molecules including p-mTORC1 and downstream molecules p-4EBP1 and p-p70S6K were increased in TCEpos group (*p* < 0.05). No significant differences were observed among the blank control group, vehicle control group, and TCEneg group (*p* > 0.05) (Figure 6). These data suggest that mTORC1 pathway was involved in TCE-induced glomerular damage.

### mTORC1 Pathway Inhibition Alleviated the Glomerular Damage in TCE Sensitization

**FIGURE 7:**
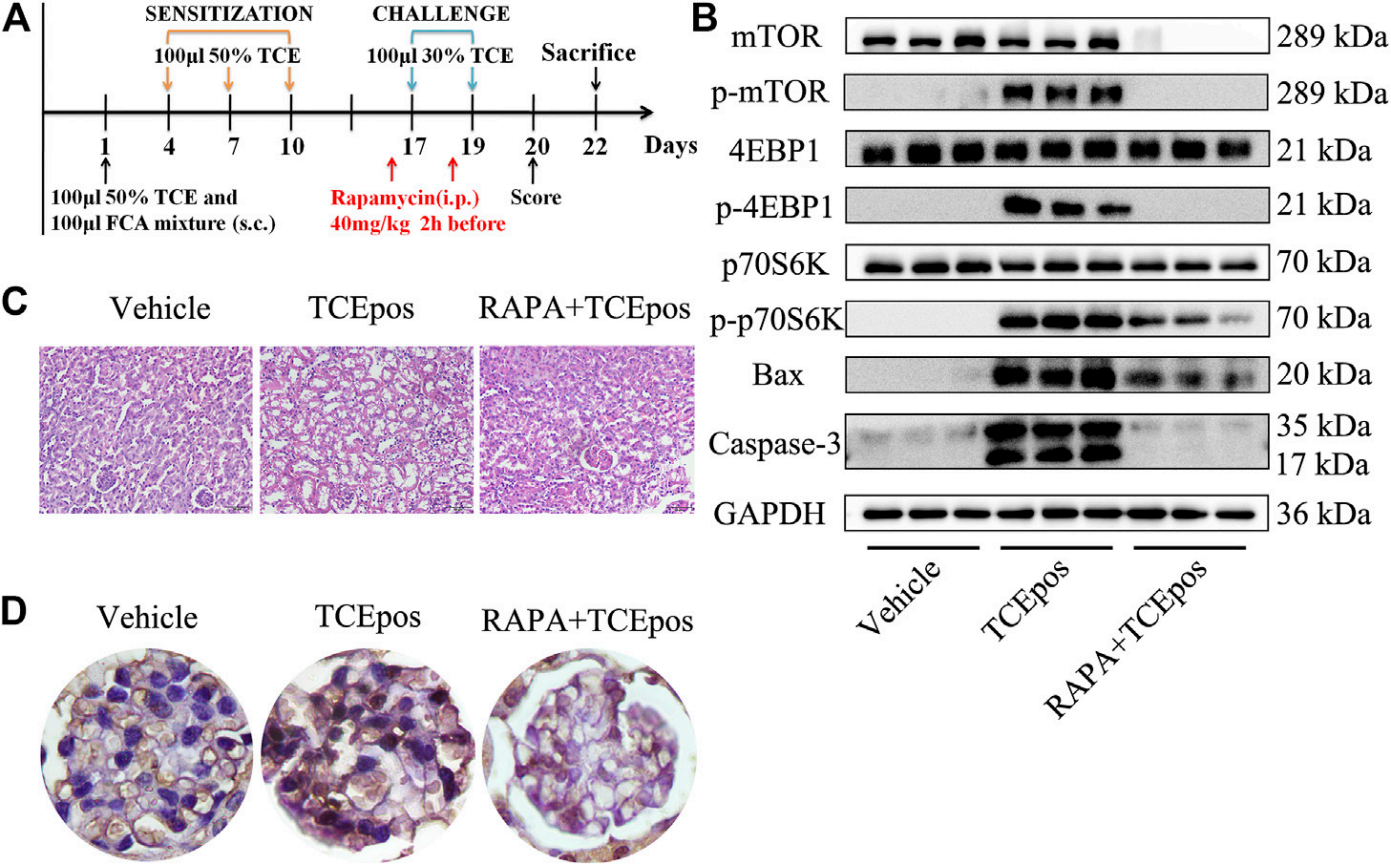
mTORC1 pathway inhibition alleviated the glomerular damage in TCE sensitization. **(A)** mTORC1 inhibitor (rapamycin, RAPA) was applied to mice by intraperitoneal injection at 40 mg/kg 2 h before each challenge. **(B)** Phosphorylation process of mTOR signaling molecules were almost blocked and the proapoptotic molecules Bax and caspase-3 were also declined in RAPA + TCEpos group according to western blot bands (*n* = 3 per group). **(C)** The pathological changes of glomeruli were improved dramatically in mice from RAPA + TCEpos group. **(D)** The representative photograph of TUNEL staining showed less dead glomerular cells in RAPA + TCEpos group.

To explore the role of hyperactive mTOR signaling in mice, rapamycin was applied by intraperitoneal injection (Figure 7A). As shown in Figure 7, the process of mTORC1 phosphorylation was completely blocked after rapamycin injection. The relative expressions of downstream p-4EBP1 and p-p70S6K were also decreased, confirmed by Western blot (Figure 7B). After pharmacological inhibition of mTOR signaling, we found that glomerular damage was improved dramatically in mice (Figure 7C). The proportion of apoptotic glomerular cells and the pro-apoptotic Bax and caspase-3 were decreased in RAPA + TCEpos group compared with TCEpos group (*p* < 0.05) (Figures 7B,D **)**. Collectively, these results suggest that hyperactive mTOR signaling contributes to glomerular cell death due to apoptosis caused by TCE sensitization.

### Cathepsin L–Mediated Hyperactive mTORC1 Signaling in TCE-Induced Glomerular Injury

**FIGURE 8:**
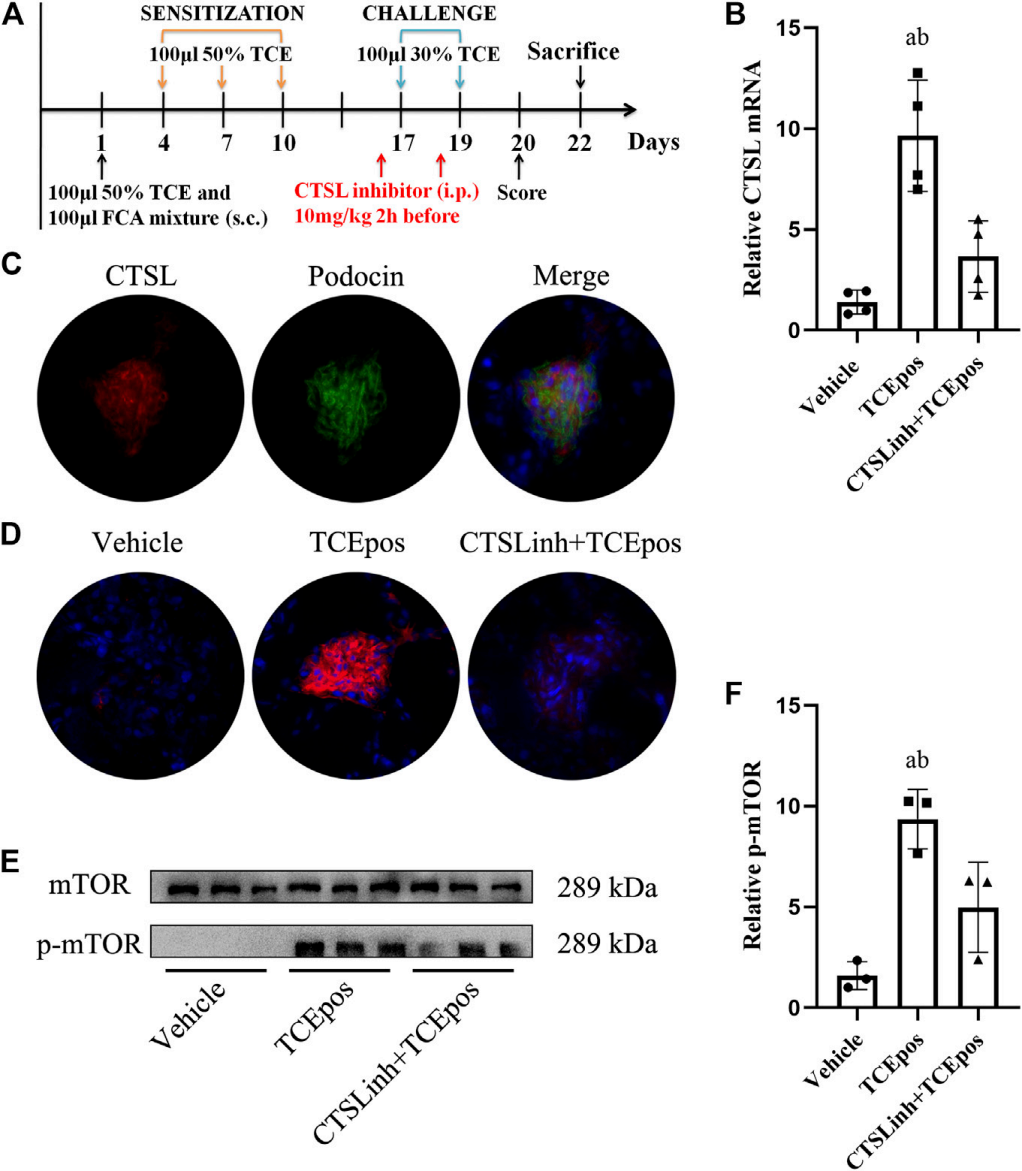
Cathepsin L (CTSL) mediated hyperactive mTORC1 signaling in TCE-induced glomerular injury. **(A)** CTSL inhibitor (CTSLinh) was applied to mice by intraperitoneal injection at 10 mg/kg 2 h before each challenge. **(B)** The relative expression of *CTSL mRNA* in vehicle group, TCEpos group, and CTSLinh + TCEpos group according to RT-PCR analysis (*n* = 4 per group). **(C)** The co-location of CTSL and podocin (a podocyte marker) in glomerulus of TCEpos group. **(D)** The expression of CTSL confirmed by immunofluorescence test (*n* = 5 per group). **(E)** Western blot bands of mTORC1 and p-mTORC1 in vehicle control group, TCEpos group, and CTSLinh + TCEpos group (*n* = 3 per group). **(F)** The relative expression of p-mTOR calculated by gray values in ImageJ software. Data are presented as mean ± SD and determined by one-way ANOVA, *p < 0.05.

To further investigate the possible regulator of mTOR signaling, we focused on the lysosomal cysteine protease cathepsin L (CTSL), which is a powerful proteolytic enzyme to regulate cell fate, including apoptosis, autophagy, proliferation, and plays a key role in metastasis (Cocchiaro et al., 2017). In line with our previous studies, we found an increase of CTSL expression in TCEpos group compared to vehicle control group (*p* < 0.05), and the expression of CTSL was mainly blocked in CTSLinh + TCEpos group (Figures 8A–D **)**.

We located the CTSL expression in glomeruli via immunofluorescence analysis with colocalization experiment. Using podocin as a hallmark of podocyte, we identified that CTSL was mainly expressed in podocyte (Figure 8C **)**. Moreover, we additionally applied CTSL inhibitor to mice and examined the effect on mTOR activation (Figure 8A **)**. We showed that p-mTOR was downregulated in CTSLinh + TCEpos group compared with TCEpos group (Figures 8E,F). These consistent results suggest that CTSL acts as a key driver for hyperactive mTOR signaling in TCE-induced glomerular damage.

## Discussion

TCE-induced immune kidney disorder is one of the most common complications of OMDT. Although the fatality rate of which is not more than hepatitis, it is closely related to the severity of disease and associated with a poor prognosis (Wei et al., 2008; Liu, 2009). Diffuse inflammation, renal dysfunction, and positive urinary proteins are often manifested in the process of TCE-induced renal damage (Liu, 2009; Xu et al., 2009). Liu (2009) reported a case after exposure to TCE for about one month with quick deterioration of renal function, displaying with a high level of BUN 23.2 mmol/L, Cre 426.4 μmol/L, and uric acid 660.2 mmol/L. Furthermore, limb edema and oliguria are also involved in some patients (Zhang et al., 2011). Consistently, our previous studies also found an increase of various proinflammatory cytokines, including TNF-α, IL-1β, IL-17, and IL-6, in TCE-hypersensitivity induced kidney damage (Wang et al., 2020; Yang et al., 2020). In this study, we focused on mTOR signaling pathway in TCE-induced inflammatory renal diseases. We measured the levels of urine nephrin to assess the damage of glomerular structure integrity. As expected, the levels of urine nephrin before clinical treatment were higher than that of after clinical treatment, which suggests a loss of nephrin from glomerulus and hence a damage of glomerular structure integrity. Consistent with findings in human cases, we also found glomerular proliferation and swelling in morphology with increased serum levels of BUN and Cre in TCE sensitization positive mice. Therefore, the above observations both in patients and mice pointed out that the normal structure and function of glomerulus are damaged to a certain degree in the process of TCE sensitization.

Acting as the special type of glomerular components, podocyte is a highly polarized epithelial cell with limited capacity to proliferate (Nagata, 2016). The structural integrity of podocyte is essential in the formation of glomerular filtration barrier and the healthy podocytes are required for the normal filtration function (Torban et al., 2019). Loss of podocytes from glomerular basement membrane presented as filtration dysfunction and even proteinuria in various kidney disorders, including diabetic nephropathy, lupus nephropathy, membranous glomerulonephritis, glomerulosclerosis, and so on (Sakhi et al., 2019). In TCE-induced glomerular disorders, podocyte hypertrophy with thickened GBM, fusion of foot processes was found under a transmission electron microscope. Furthermore, we also found that a decline of integrin β1 which is the transmembrane anchoring molecule for podocyte adhesion to GBM (Perico et al., 2016; Sawada et al., 2016) can likely be a reason for the loss of podocyte. A decrease of α-actinin-4 which is a key molecule for maintain the healthy cytoskeleton may explain the foot process fusion and effacement. Collectively, decline of nephrin, α-actinin-4, and integrin β1 may indicate that podocyte damage participates in the renal dysfunction caused by TCE sensitization.

Furthermore, we also assessed apoptosis of glomerular cells in TCE-induced glomerular damage. Bax and caspase-3 are known to promote the apoptotic progression, whereas Bcl-2 works conversely (Ma et al., 2020). The balance between the pro-apoptotic Bax, caspase-3 and anti-apoptotic Bcl-2 determines cell survival or death (Pal et al., 2016). We showed that the proportion of both apoptotic glomerular cells and the pro-apoptotic Bax and caspase-3 were increased in TCE positive sensitized mice. Thus, these coherent data suggest that glomerular apoptosis also contribute to the TCE-induced kidney damage.

To uncover the molecular mechanism of podocyte damage and glomerular apoptosis, we focused on mTORC1 signaling which has proven to be critical in glomerular diseases via promoting cellular growth and metabolism (Zschiedrich et al., 2017; Lei et al., 2018). However, hyperactivated mTORC1 signaling was considered to be the main causes of podocyte hypertrophy, foot process fusion, and ultimately cell death (Puelles et al., 2019). During activation of mTORC1 signaling, two downstream molecules, p70S6K and 4EBP1, are phosphorylated (Hall et al., 2018). In this study, the over-activation of mTORC1 signaling was evidenced by increased expression of p-mTOR, p-p70S6K, and p-4EBP1. To further elucidate the role of mTORC1 signaling in TCE-induced glomerular damage, we applied rapamycin by intraperitoneal injection to TCE positive sensitized mice. After rapamycin pretreatment, the hyperactive mTORC1 signaling was effectively minimized in mice glomerulus. Furthermore, we found that glomerular apoptosis and structural destruction were also ameliorated after pharmacological inhibition of hyperactive mTORC1 signaling. Collectively, we identified that hyperactive mTORC1 signaling caused glomerular damage and rapamycin reversed cell injuries in TCE-induced glomerular damage.

Furthermore, we explored the possible upstream driver of mTORC1 signaling in TCE-induced glomerular damage. Studies have shown that CTSL could cleave the key elements of normal podocyte architecture and cause podocyte reorganization, foot process effacement, and even proteinuria (Reiser et al., 2010). In addition, a role of CTSL in regulation of mTORC1 signaling activation via cleaving intracellular complement 3 has been reported (Satyam et al., 2017). Our previous studies have also found that over-expressed CTSL could aggravate kidney damage via activating intracellular complement system and inducing endothelin-1 signaling to promote the release of inflammatory cytokines (Wang et al., 2020; Yang et al., 2020). In this study, we further located CTSL to podocyte and investigated whether over-expressed CTSL played a role in mTORC1-mediated glomerular damage. We found that a downregulation of mTOR phosphorylation after CTSL inhibitor pretreatment. Therefore, these results suggest that CTSL could be a driver for hyperactive mTORC1 signaling in TCE-induced glomerular damage.

However, there are some limitations in the present study. First, only 6 OMDT cases were enrolled in the present study due to the fact that OMDT is a rare but life-threatening disorder with a prevalence of less than 1% among TCE-exposed workers and the overall incidence was less than 1 case/million adults/year (Lin et al., 2019). Second, only female mice were used to establish the mouse model of TCE skin sensitization. Two main reasons were considered. On the one hand, there is no evidence of sex-based differences in TCE-sensitized workers, and both male and female exposure workers are susceptible to TCE sensitization. On the other hand, female mice have been shown to exhibit more consistent pronounced inflammatory response than male mice with elevated levels of CD4^+^ T-cells and relevant proinflammatory cytokine, and thereby are more widely used in animal models of allergic diseases, like asthma, atopic dermatitis, and allergic rhinitis (Melgert et al., 2005). Third, no *in vitro* sensitization experiment was performed, as how to maintain the *in situ* environment of TCE sensitization *in vitro* is unresolved. Finally, the mouse model of TCE sensitization is an appropriate substitute of guinea pig maximization test (GPMT), which is a classical method for evaluating skin sensitization by various chemicals including TCE, but it’s not widely used due to lack of relevant antibodies and reagents for guinea pigs.

In conclusion, our results highlight that glomerular damage involved in TCE-induced immune kidney disorder in which hyperactive mTORC1 signaling contributed to podocyte loss, hypertrophy, and glomerular apoptosis in TCE-induced glomerular injuries. Furthermore, our data identify a role of CTSL in the regulation of over-activated mTORC1 signaling in TCE sensitization positive mice and the associate glomerular damage.

## Data Availability

The original contributions presented in the study are included in the article/Supplementary Material; further inquiries can be directed to the corresponding authors.
